# Clinical, sociodemographic and environmental factors impact post-COVID-19 syndrome

**DOI:** 10.7189/jogh.12.05029

**Published:** 2022-08-09

**Authors:** Juliana Carvalho Ferreira, Tiana C Lopes Moreira, Adriana Ladeira de Araújo, Marta Imamura, Rodolfo F Damiano, Michelle L Garcia, Marcio VY Sawamura, Fabio R Pinna, Bruno F Guedes, Fabio A Rodrigues Gonçalves, Marcio Mancini, Emmanuel A Burdmann, Demóstenes Ferreira da Silva Filho, Jefferson Lordello Polizel, Ricardo F Bento, Vanderson Rocha, Ricardo Nitrini, Heraldo Possolo de Souza, Anna S Levin, Esper G Kallas, Orestes V Forlenza, Geraldo F Busatto, Linamara R Batistella, Carlos R Ribeiro de Carvalho, Thais Mauad, Nelson Gouveia, the HCFMUSP COVID-19 Study Group, Tarcisio E.P. Barros-Filho, Edivaldo M. Utiyama, Aluisio C. Segurado, Beatriz Perondi, Anna Miethke-Morais, Amanda C. Montal, Leila Harima, Solange R.G. Fusco, Marjorie F. Silva, Marcelo C. Rocha, Izabel Marcilio, Izabel Cristina Rios, Fabiane Yumi Ogihara Kawano, Maria Amélia de Jesus, Juliana C. Ferreira, Carlos R de Carvalho, Esper G Kallas, Anna Sara S. Levin, Heraldo Possolo de Souza, Maria Cristina Peres Braido Francisco, Carolina Mendes do Carmo, Clarice Tanaka, Maura Salaroli Oliveira, Thaís Guimarães, Carolina dos Santos Lázari, Marcello M.C. Magri, Julio F.M. Marchini, Alberto José da Silva Duarte, Ester Sabino, Silvia Figueiredo Costa

**Affiliations:** 1 Divisao de Pneumologia, Instituto do Coração, Hospital das Clinicas HCFMUSP, Faculdade de Medicina, Universidade de São Paulo, SP, Brasil; 2 Intensive Care Unit, AC Camargo Cancer Center, São Paulo, Brasil; 3 Departamento de Patologia, LIM/05- Laboratório de Poluição Atmosférica Experimental, Hospital das Clínicas da Faculdade de Medicina da Universidade de São Paulo HCFMUSP, São Paulo, SP, Brasil; 4 Diretoria Executiva dos LIMs, Faculdade de Medicina da Universidade de São Paulo HCFMUSP, São Paulo, SP, Brasil; 5 Instituto de Medicina fisica e Reabilitação do Hospital das Clinicas, Departamento de Medicina Legal, Etica Médica e Medicina Social e do Trabalho, Faculdade de Medicina da Universidade de São Paulo HCFMUSP, São Paulo, SP, Brasil; 6 Departamento e Instituto de Psiquiatria, Hospital das Clínicas da Faculdade de Medicina da Universidade de São Paulo HCFMUSP, São Paulo, SP, Brasil; 7 Departamento de Radiologia, Hospital das Clínicas da Faculdade de Medicina da Universidade de São Paulo HCFMUSP, São Paulo, SP, Brasil; 8 Departamento de Oftalmologia e Otorrinolaringologia, Faculdade de Medicina da Universidade de São Paulo HCFMUSP, São Paulo, SP, Brasil; 9 Departamento de Neurologia, Faculdade de Medicina da Universidade de São Paulo HCFMUSP, São Paulo, SP, Brasil; 10 Departamento de Cardiopneumologia, Laboratório de Cirurgia Cardiovascular e Fisiopatologia da Circulação, Faculdade de Medicina da Universidade de São Paulo HCFMUSP, São Paulo, SP, Brasil; 11 Unidade de Obesidade e Síndrome Metabólica, Disciplina de Endocrinologia e Metabologia, Hospital das Clínicas da Faculdade de Medicina da Universidade de São Paulo HCFMUSP, São Paulo, SP, Brasil; 12 Departamento de Clínica Médica, LIM/12 - Laboratório de Pesquisa Básica em Doenças Renais, Disciplina de Nefrologia, Faculdade de Medicina da Universidade de São Paulo, São Paulo, SP, Brasil; 13 Departamento de Ciências Florestais-ESALQ/USP, Laboratório de Silvicultura Urbana, Universidade de São Paulo, Piracicaba, SP, Brasil; 14 Departamento de Ciências Florestais-ESALQ/USP, Laboratório de Métodos Quantitativos, Universidade de São Paulo, Piracicaba, SP, Brasil; 15 Divisão de Otorrinolaringologia, Hospital das Clínicas da Faculdade de Medicina da Universidade de São Paulo HCFMUSP, São Paulo, SP, Brasil; 16 Serviço de Hematologia, Hemoterapia e Terapia Celular, Divisão de Clínica Médica I do ICHC, Hospital das Clínicas da Faculdade de Medicina da Universidade de São Paulo HCFMUSP, São Paulo, SP, Brasil; 17 Departamento de Clínica Médica, Disciplina de Emergências Clínicas, Hospital das Clínicas da Faculdade de Medicina da Universidade de São Paulo HCFMUSP, São Paulo, SP, Brasil; 18 Departamento de Moléstias Infecciosas e Parasitárias, Hospital das Clínicas da Faculdade de Medicina da Universidade de São Paulo HCFMUSP, São Paulo, SP, Brasil; 19 Departamento e Instituto de Psiquiatria, Laboratório de Neurociências - LIM-27, Hospital das Clínicas da Faculdade de Medicina da Universidade de São Paulo HCFMUSP, São Paulo, SP, Brasil; 20 Departamento de Medicina Preventiva, Faculdade de Medicina da Universidade de São Paulo HCFMUSP, São Paulo, SP, Brasil

## Abstract

**Background:**

Sociodemographic and environmental factors are associated with incidence, severity, and mortality of COVID-19. However, little is known about the role of such factors in persisting symptoms among recovering patients. We designed a cohort study of hospitalized COVID-19 survivors to describe persistent symptoms and identify factors associated with post-COVID-19 syndrome.

**Methods:**

We included patients hospitalized between March to August 2020 who were alive six months after hospitalization. We collected individual and clinical characteristics during hospitalization and at follow-up assessed ten symptoms with standardized scales, 19 yes/no symptoms, a functional status and a quality-of-life scale and performed four clinical tests. We examined individual exposure to greenspace and air pollution and considered neighbourhood´s population density and socioeconomic conditions as contextual factors in multilevel regression analysis.

**Results:**

We included 749 patients with a median follow-up of 200 (IQR = 185-235) days, and 618 (83%) had at least one of the ten symptoms measured with scales. Pain (41%), fatigue (38%) and posttraumatic stress disorder (35%) were the most frequent. COVID-19 severity, comorbidities, BMI, female sex, younger age, and low socioeconomic position were associated with different symptoms. Exposure to ambient air pollution was associated with higher dyspnoea and fatigue scores and lower functional status.

**Conclusions:**

We identified a high frequency of persistent symptoms among COVID-19 survivors that were associated with clinical, sociodemographic, and environmental variables. These findings indicate that most patients recovering from COVID-19 will need post-discharge care, and an additional burden to health care systems, especially in LMICs, should be expected.

By November 2021, COVID-19 caused more than 250 million cases, with more than five million deaths worldwide [1]. Cohort studies of hospitalized patients identified risk factors for poor outcomes and revealed mortality rates ranging from 25% to 49% [2-5]. In addition, among recovering patients, reports of persistent symptoms emerged, first by patients on social media and later defined as “post-COVID-19 syndrome” when symptoms persist after 12 weeks [6].

Recent data show that persistent symptoms affect more than half of recovering patients [7-9]. Fatigue, dyspnoea, sleep disturbances [8], and psychiatric and cognitive symptoms [10] are the most common. Patients that had more severe forms of COVID-19 [8] and females [7,8] are at higher risk of persistent symptoms. Our knowledge on the mechanisms involved, other potential risk factors and treatment options for these symptoms are still limited.

Besides clinical features, there is evidence that sociodemographic and environmental risk factors can influence the incidence, severity, and mortality of COVID-19. Socioeconomic disparities were associated with differences in mortality in Brazil [3], and in Sao Paulo, low-income communities had higher risk of hospitalization and death [10]. Population density also appears to have an impact on morbidity and mortality [11]. Furthermore, the risk of infection and death is not only higher in the poorest neighbourhoods, but also among the poorest individuals [12], suggesting an interaction between individual and contextual socioeconomic factors [13].

Evidence also exists linking air pollution with increased COVID-19 incidence and mortality [14]. Chronic exposure to air pollution impairs lung function and increases vulnerability to respiratory diseases, particularly infections [15]. On the other hand, studies have indicated that exposure to greenspace may play a role in reducing risk of mortality in COVID-19 [16]. This beneficial effect might be related to reduced exposure to air pollution, greater diversity of microbial exposure and enhanced immunity [17].

Sao Paulo was the epicentre of COVID-19 cases in Brazil and, like other megacities of low-and middle-income countries (LMIC), copes with important socioeconomic disparities, irregular distribution of greenspace, high levels of air pollution and complex urban arrangements [18], offering an appropriate setting to evaluate post-COVID-19 syndrome. We hypothesized that, in this city, not only individual but sociodemographic, and environmental factors could be associated with the persistence of symptoms in COVID-19. Identifying such factors is important in order to create health policies to prevent or mitigate long term health consequences and provide patient-centred care for a growing number of survivors worldwide. Therefore, we designed a cohort study of hospitalized COVID-19 survivors, to describe clinical characteristics and persistent symptoms after six months of hospitalization and identify clinical, sociodemographic, and environmental factors associated with post-COVID-19 syndrome.

## METHODS

### Study design and location

This is a cohort study conducted at Hospital das Clínicas from the University of Sao Paulo Medical School (HCFMUSP). The study protocol was published elsewhere [19]. In brief, the largest building of an academic hospital was turned into a dedicated facility for COVID-19 patients, transferred from health services across the metropolitan area of Sao Paulo, where approximately 23 million people live. A total of 900 beds were made available, and over 3000 patients were admitted from March 30th to August 31st, 2020.

### Study population

We aimed at very broad inclusion criteria, hoping to include as many survivors of the first wave of COVID-19 as possible. Thus, the inclusion criteria were survival six months after hospitalization, hospital stay of at least 24 hours, age >18 years and confirmed COVID-19. Hospital stays shorter than 24 hours were not included because for those patients we did not have complete baseline data. Exclusion criteria were nosocomial COVID-19 infection, given that they were admitted to the hospital for other severe acute conditions, previous diagnosis of dementia or end-stage cancer, subjects living in long-term care facilities or with insufficient mobility to leave home after six months of hospital discharge, since the performance of some of the tests and scales would not be feasible for such patients, suspected reinfection at the time of follow-up and refusal to participate in the study.

Patients were invited to participate in the study by telephone and those who accepted were scheduled for in-person visits. Visits were scheduled for within three weeks of the six-month mark after hospitalization. However, patients who agreed to participate but were unavailable at that window had their appointments rescheduled and were evaluated later (see Appendix S1 in the **Online Supplementary Document**).

This study integrates the results of several research projects led by health specialist teams within HCFMUSP. All projects were approved by the Ethics Committee (approval numbers: 4.270.242, 4.502.334, 4.524.031, 4.302.745 and 4.391.560). Informed consent was obtained from all participants.

### Sociodemographic variables

Age, sex, years of education, socioeconomic position and race were collected. Socioeconomic position was measured by a standardized questionnaire validated for the Brazilian population which classified individuals in seven categories (A-most affluent, B1, B2, C1, C2, D and E) [20]. Race was self-declared, using the official Brazilian categories (white, mixed, black, Asian, indigenous). (More details in Appendix 1 the **Online Supplementary Document**).

We also collected information on population and average per capita income for each participant's neighbourhood. We divided the population by the area of each neighbourhood to compute the population density. The average income (in US$) was used as an indicator of the socioeconomic conditions of the neighbourhood.

### Clinical assessments

We registered comorbidities at hospital admission with the Charlson comorbidity index [21]. Post COVID-19-symptoms were obtained with standardized scales, when available, or by direct question and a yes/no answer. We assessed 10 symptoms measured by standardized scales, 19 yes/no symptoms, a functional status scale, a quality-of-life scale, and performed four tests (spirometry, chest x-ray, 1-minute sit-to-stand test and hand grip strength test).

We present results for all symptoms, scales and tests performed but focused on four outcomes representative of the post-COVID-19 syndrome and which were measured with standardized scales to minimize bias: dyspnoea, assessed with the Medical Research Council (MRC) scale [22]; fatigue, assessed with the Functional Assessment of Chronic Illness Therapy-Fatigue Scale (FACIT) [23]; anxiety and depression, assessed with the Hospital Anxiety and Depression scale (HADS) [24]; and functional status, assessed with the Post-COVID-19 Functional Status (PCFS) Scale [25].

A full list detailing evaluations and instruments is presented in Table S1 in the **Online Supplementary Document**.

### Environmental variables

To estimate exposure to greenspace and air pollution, each participant´s residential address was georeferenced and a 300 m buffer area around each address was created. We used satellite images of the Sao Paulo metropolitan region for 2018 and 2020, to classify and quantify the land covered by greenspace. The 300 m buffer corresponds to approximately five min walking distance from the household and is recommended by the WHO [26].

Gridded satellite estimates of annual mean levels of air pollution (PM_2.5_ – particulate matter up to 2.5μm diameter) were obtained for 2018, and we averaged the gridded values for the same buffer areas and these values were assigned to each participant. Further details of the greenspace and air pollution exposure assessment are in Appendix 1 in the **Online Supplementary Document**.

### Data collection

Study data were collected and managed using a secure, web-based platform (REDCap – Research Electronic Data Capture) [27]. The results are reported in accordance with the Strengthening The Reporting of Observational Studies in Epidemiology (STROBE) guidelines [28].

### Statistical analysis plan

We did not perform a sample size estimation, and instead aimed at recruiting as many survivors of the large cohort of COVID-19 hospitalized patients as possible, using broad inclusion criteria, minimizing exclusion criteria and implementing a well-organized recruitment strategy.

We describe the prevalence of symptoms and present the results of several clinical evaluations at follow up. Categorical variables are expressed as count and percentage, and continuous variables, as mean and standard deviation, or median and interquartile range (IQR) as appropriate. We created histograms to visualize the variable´s distribution followed by the Shapiro-Wilk test to check for normality.

We conducted a multilevel regression analysis using a generalized linear mixed model with random intercepts at the neighbourhood level to identify whether clinical, sociodemographic, and environmental factors were associated with the four selected outcomes. In the multilevel models, individual variables were included in the first level and the contextual characteristics of neighbourhoods (population density and per capita income) in the second level. The variance inflation factor (VIF) was used to check the multicollinearity of the variables.

We first examined each variable in univariate models, then we fitted multivariate models with variables that showed an association with at least one of the outcomes in the univariate analysis. We retained the two contextual variables (population density and neighbourhood socioeconomic conditions) and the environmental exposures (air pollution and greenspace) in the multivariate models regardless of their significance in the univariate models. We dropped the variable education as our indicator of socioeconomic position already included education. We present the adjusted estimates of changes in each outcome variable for a unit change in each predictor.

All tests were two-tailed with a significance level of 0.05 and performed using the Rstudio software [29]. Missing data was minimal for clinical characteristics and no imputation methods were used.

### Ethics approval and consent to participate

This study integrates the results of several research projects within HCFMUSP. All projects were approved by the HCFMUSP Ethics Committee (approval numbers: 4.270.242, 4.502.334, 4.524.031, 4.302.745 and 4.391.560). Informed consent was obtained from all participants.

## RESULTS

**Figure 1:**
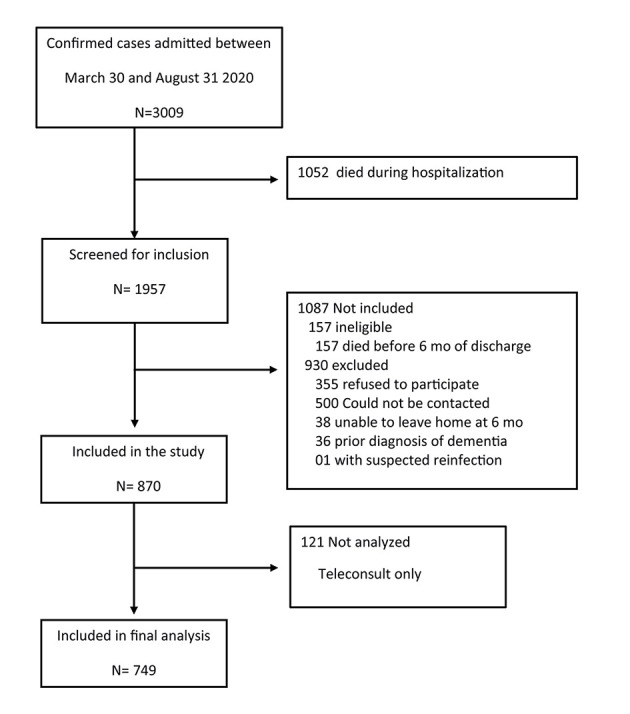
Study participant flow. Legend: Flow of potentially eligible participants in the study, and final numbers included and analysed.

Of 3009 patients with COVID-19 who were admitted to the hospital between March 30 and August 31, 2020, 1957 survived hospitalization and 749 were included in the study (**Figure 1**). The median number of days after discharge for follow-up assessments was 200 (IQR = 185-235) days.

Comparisons between patients who participated in the study and those excluded are shown in Table S2 in the **Online Supplementary Document**. While demographic characteristics were comparable, participants had higher body mass index (BMI), more previous hypertension, and more severe disease, indicated by longer duration of hospitalization and more need for ICU and intubation during hospitalization.

**Table 1** shows sociodemographic and clinical characteristics during hospitalization for all participants. Approximately half were male, with age of 55 ± 14 years-old, predominantly white, with low education, and middle to low socioeconomic position. Almost 60% were admitted to the ICU during hospitalization.

**Table 1:** Baseline and hospitalization characteristics of participants

Characteristic	All (n = 749)
**Age, y, mean ± SD**	55 ± 14
Male	397 (53%)
Body mass index, kg/m^2^, median (IQR)	31 · 1 (27 · 5-36 · 6)
Race*	
White	342 (47%)
Asian	10 (1%)
Mixed	273 (37%)
Black	102 (14%)
Indigenous	7 (1%)
Education	
<4 y	265 (36%)
4-8 y	142 (19%)
8-12 y	202 (27%)
>12 y	134 (18%)
**Socioeconomic position^†^**	
A+B1+B2 (high)	196 (27%)
C1+C2 (medium)	470 (64%)
D+E (low)	73 (10%)
Smoking history, yes	284 (38%)
Charlson comorbidity index, median (IQR)	3 (2-4)
Duration of symptoms at admission, median (IQR), d	8 (6-11)
Acute renal failure during hospitalization	315 (42%)
ICU stay	445 (59%)
Intubation	305 (41%)
Duration of hospitalization, median (IQR), d	12 (7-23)

SD – standard deviation, IQR – interquartile range, y – years

Data are presented as counts (percentage) unless otherwise stated.

*The categories represent the Brazilian official race categories.

^†^Brazilian official socio-economic classes, based on household assets, access to public services, and educational level of the head of the family, with A being the higher income class and E the lower income class. We collapsed categories A+B1+B2 (high), C1+C2 (medium) and D+E (low) for the present analysis. Body mass index missing for 9 (1%) patients. Race missing for 15 (2%) patients. Education level missing for 6 (1%) patients. Socioeconomic position missing for 10 (1%) patients. Charlson comorbidity index missing for 30(4%) patients. Smoking history missing for 6 (1% patients). PM_2.5_ missing for 1 (0%) patient. Duration of symptoms missing for 1 (0%) patient. Acute renal failure was defined and staged according to the KDIGO definition using serum creatinine criteria.

At follow-up, 618 (83%) had at least one of the ten symptoms measured with standardized instruments (Figure S1 in the **Online Supplementary Document**), and the median number of symptoms was 2 (IQR = 1-5). **Table 2** shows the results of scales and tests performed at follow-up. Pain, fatigue, posttraumatic stress disorder (PTSD), and memory impairment were the most common symptoms. Diagnostic tests showed impairment in muscle strength for almost two thirds and pulmonary function abnormalities in one third of patients. Table S3 in the **Online Supplementary Document** shows the prevalence of additional symptoms.

**Table 2:** Symptoms, scales, and test results at follow-up*

	All (n = 749)	Percent abnormal
**Objective symptoms**		
Muscle/joint pain, VAS (0-100) (abnormal if ≥65)	40 (10-65)	41%
Fatigue, score (0-52) (abnormal if ≤39)	42 (33-47)	38%
Posttraumatic stress disorder, score (0-85), (abnormal if ≥30)	24 (19-36)	35%
Memory impairment, score (0-14) (abnormal if ≥7)	4 (1-8)	35%
Insomnia, score (0-28) (abnormal if ≥8)	6 (2-11)	32%
Dyspnoea, score (0-5), (abnormal ≥2)	1 (0-2)	30%
Anxiety, points (0-21) (abnormal if >8)	5 (2-9)	26%
Loss of taste, VAS (0-100), (abnormal if ≤80)	100 (85-100)	23%
Depression, points (0-21) (abnormal if >8)	3 (1-7)	22%
Loss of smell, VAS (0-100), (abnormal if ≤80)	100 (84-100)	21%
**Scales**		
Post COVID functionality, points (0-4), (abnormal if ≥2)	1 (0-2)	32%
Quality of Life, VAS (0-100)	80 (60-90)	NA
**Diagnostic tests**		
Muscle strength, kgf, (abnormal if <25% age percentile)	19 (10-28)	64%
SpO2 at rest, % (abnormal if <92%)	97 (95-98)	7%
SpO2 at the end of sit-to-stand test % (abnormal if decrease ≥4% from baseline)	96 (95-98)	10%
Forced vital capacity, % predicted (abnormal if <80% of predicted value)	84 (74-94)	32%
Abnormal X Ray (according to radiologist)	NA	29%

SpO2 – peripheral saturation of oxygen measured at rest and at the end of the sit-to-stand test, HADS – hospital anxiety and depression scale

*Values are median (interquartile range) and percentage of the total population with abnormal results according to cut off values shown in Table S1 in the **Online Supplementary Document**. Scales and instruments used to measure each symptom or test are shown in Table S1 in the **Online Supplementary Document**. Dyspnoea was missing for 8 participants; SpO2 at rest was missing for 3 participants; sit-to-stand test was missing for 73 participants; forced vital capacity was missing for 108 participants; x-ray was missing for 122 participants; HADS was missing for 78 participants; posttraumatic stress disorder scale was missing for 14 participants; post COVID functionality was missing for 3 participants; insomnia was missing for 3 participants; fatigue was missing for 3 participants; muscle/joint pain was missing for 15 participants; loss of smell was missing for 22 participants; loss of taste was missing for 34; muscle strength was missing for 23; quality of life was missing for 4.

To examine whether timing of evaluation at follow-up had an impact on our findings, we compared patients evaluated before 200 days of hospitalization (the median) and those evaluated after 200 days. We found that patients evaluated later were less likely to have developed acute renal failure during hospitalization, and had shorter ICU and hospital stay (Table S4 in the **Online Supplementary Document**). They also had a higher prevalence of anxiety, depression, dyspnoea, severe muscle/joint pain, and loss of smell at follow-up (Table S5 in the **Online Supplementary Document**).

**Figure 2:**
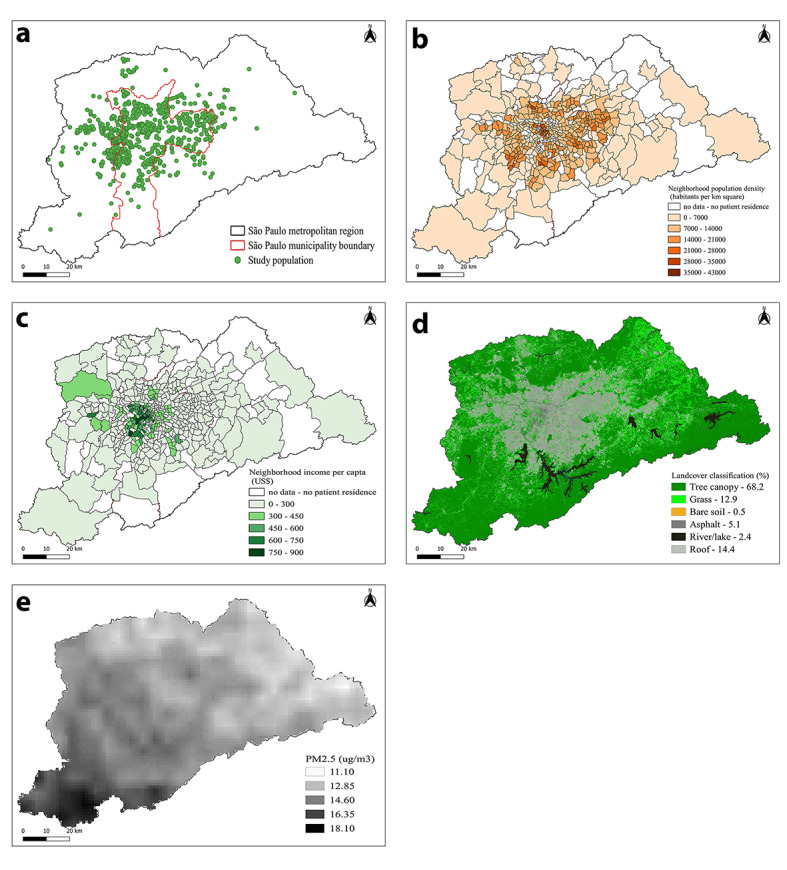
Patients place of residence, sociodemographic and environmental characteristics of the Sao Paulo Metropolitan Region. Legend: Maps of the Sao Paulo Metropolitan Region, comprising 39 municipalities and where approximately 23 million people live. a) green dots represent the origin (residency) of each participant. The red line represents the borders of the city of Sao Paulo; b) population density in each neighbourhood; c) the average per capita income of the neighbourhoods; d) the distribution of greenspace; e) air pollution levels.

**Figure 2**, Panel A, shows that participants came from several neighbourhoods and different cities across the metropolitan region of Sao Paulo. The maps also show the population density in each neighbourhood (**Figure 2**, Panel B), the average per capita income of the neighbourhoods (**Figure 2**, Panel C), the distribution of greenspace (**Figure 2**, panel D), and air pollution levels (**Figure 2**, Panel E). The median exposure to PM_2,5_ of participants was 14.4μg/m^3^ (range = 12.5-20.8) and the median greenspace exposure in the 300m buffers was 17.0% (range = 2.2%-99.7%).

Univariate analysis (Table S6 in the **Online Supplementary Document**) indicated that sociodemographic characteristics were significantly associated with the outcomes examined. Comorbidities, BMI, and indicators of COVID-19 severity also showed to be predictors of symptom persistence. Smoking and race were not associated with the selected outcomes. Exposure to air pollution showed a statistically significant association only with dyspnoea, but the direction of the associations was consistent for all outcomes. On the other hand, exposure to greenspace showed an inconsistent pattern, acting either as a protective or risk factor, but was not statistically associated with the outcomes.

**Table 3** presents the multilevel linear multivariate estimates for sociodemographic, clinical, and environmental factors associated with each predefined outcome. Female sex was associated with increased dyspnoea, lower functional status, increased fatigue, and higher anxiety and depression scores. Younger age was associated with higher anxiety and depression, while lower socioeconomic position was associated with increased dyspnoea, increased fatigue, and worse functional status.

**Table 3:** Regression estimates for sociodemographic, clinical, and environmental factors associated with selected persistent symptoms and functional scale in patients with COVID-19 at follow-up

		Dyspnoea		Fatigue		Functional status		Anxiety/depression	
**Estimate (95% CI)**	***P-*value**	**Estimate (95% CI)**	***P-*value**	**Estimate (95% CI)**	***P-*value**	**Estimate (95% CI)**	***P-*value**
Sex	Female	**-**		-		**-**		**-**	
	Male	-0.39 (-0.55, -0.23)	**<0.001**	4.79 (3.37-6.20)	**<0.001**	-0.39 (-0.56, -0.23)	**<0.001**	-4.93 (-6.24, -3.61)	**<0.001**
Age		0.00 (-0.01, 0.01)	0.93	0.06 (-0.01, 0.13)	0.08	0.00 (-0.01, 0.01)	0.76	-0.08 (-0.14, 0.01)	**0.02**
Socioeconomic position	High	-		-		-		**-**	
	Medium	0.31 (0.13-0.50)	**<0.001**	-0.07 (-1.70, -1.56)	0.94	0.10 (-0.09, 0.29)	0.29	0.71 (-0.81, 2.22)	0.36
	Low	0.59 (0.30-0.88)	**<0.001**	-2.66 (-5.27, -0.06)	**0.05**	0.38 (0.08-0.69)	**0.01**	1.93 (-0.50, 4.35)	0.12
Charlson score		0.08 (0.02-0.14)	**0.01**	-0.87 (-1.39, -0.36)	**<0.001**	0.09 (0.03-0.15)	**0.01**	0.18 (-0.30, 0.66)	0.47
Body mass index		0.02 (0.01-0.03)	**<0.001**	-0.06 (-0.16, 0.03)	0.20	0.00 (-0.01, 0.01)	0.56	0.03 (-0.06, 0.12)	0.51
Intubation	No	**-**		**-**		**-**		**-**	
	Yes	-0.12 (-0.31, 0.07)	0.21	2.11 (0.45-3.78)	**0.01**	-0.11 (-0.31, 0.08)	0.25	-1.81 (-3.35, -0.26)	**0.02**
Length of hospital stay		0.00 (-0.01, 0.01)	0.76	-0.04 (-0.09, -0.00)	**0.05**	0.01 (0.01-0.02)	**<0.001**	0.00 (-0.04, 0.04)	0.85
PM_2.5_ (air pollution)		0.16 (0.01-0.32)	**0.03**	-1.43 (-2.73, -0.12)	**0.03**	0.16 (0.01-0.31)	**0.03**	0.50 (-0.71, 1.72)	0.42
Greenspace		0.00 (-0.01, 0.01)	0.66	0.00 (-0.05, 0.05)	0.92	0.00 (-0.01, 0.01)	0.93	0.00 (-0.05, 0.05)	0.92
Per capita income		0.00 (-0.00, 0.00)	0.12	0.00 (-0.00, 0.00)	0.16	0.00 (-0.00, 0.00)	0.45	0.00 (-0.00, 0.00)	0.82
Population density		0.00 (-0.00, 0.00)	0.80	0.01 (-0.01, 0.02)	0.34	0.00 (-0.00, 0.00)	0.48	0.00 (-0.01, 0.01)	0.68

95% IC – 95% confidence interval

We collapsed socioeconomic position categories A+B1+B2 (high), C1+C2 (medium) and D+E (low) for the present analysis. Estimates are coefficients from the multilevel regression. *P* values obtained with a multilevel model.

More comorbidities at admission were associated with increased dyspnoea, increased fatigue, and lower functional status at follow-up. BMI was associated with increased dyspnoea and intubation during hospitalization with decreased fatigue and with lower scores for anxiety and depression. Hospital length of stay was associated with increased fatigue and worse functional status (**Table 3**).

Neighbourhood per capita income and population density, and individual exposure to greenspace did not show any statistically significant association with any of the outcomes studied while exposure to ambient air pollution was associated with increased dyspnoea, lower functional status, and increased fatigue.

## DISCUSSION

In this observational study of COVID-19 survivors hospitalized in the largest public tertiary care hospital in Sao Paulo, Brazil, during the first surge of cases in 2020, we found that 83% of patients persisted with symptoms 6 months after hospitalization. The most prevalent symptoms were joint/muscle pain and fatigue. Diagnostic tests showed that two thirds of the patients had impairment of muscle strength and almost one third had pulmonary function abnormalities. In addition, we found that clinical, sociodemographic, and environmental characteristics were associated with post-COVID-19 syndrome.

The prevalence of persistent symptoms in our study is higher than what has been found elsewhere. For example, a large Chinese and a French cohort reported prevalence of 76% and 51% respectively [8,9]. Our higher estimate may be due to the severity of the acute disease among our participants, as noted by the high rate of ICU admission and need for mechanical ventilation. In addition, it may reflect a degree of selection bias since participants in our study had higher severity of disease compared to those who did not participate. However, a high prevalence of long-term health consequences after COVID-19 should not be a surprise. Previous studies showed that a significant proportion of survivors of acute respiratory distress syndrome (ARDS) have persistent physical disability or cognitive impairment one year after discharge [30], with fatigue, dyspnoea and PTSD being commonly reported. Yet, survivors of ARDS typically endure several days of hospitalization, while persistent symptoms in COVID-19 have been reported in cohorts including mild cases [31]. This suggests that a large number of recovered cases of COVID-19 are expected to present persistent symptoms, which will require specialized health attention, causing a significant burden on health care services.

The high prevalence of symptoms after hospital discharge is probably influenced by peculiarities of the health care system in Sao Paulo, and in LMICs in general. It is known that the burden of critical illness is higher in LMICs [32] and large epidemiological studies found an elevated mortality for patients under mechanical ventilation [33] or with sepsis [34] in Brazil. The interplay between structural deficiencies in the health care system, more severe disease at admission [5], lower access to health care before and after the COVID-19 hospitalization, and socioeconomic factors may lead to worse overall health and exacerbate the impact of the COVID-19 after hospital discharge.

Our observed prevalence of joint/muscle pain, fatigue, dyspnoea, and of neuropsychiatric symptoms, such as PTSD, memory impairment, sleep disturbances, anxiety and depression were slightly higher or comparable to previous studies [7-9]. Post-COVID-19 functional status was abnormal in approximately one third of participants, similar to what was previously observed [35] and underscores the impact in daily life of COVID-19 survivors several months after hospital discharge.

The multivariate analysis identified several factors associated with the persistence of symptoms, including clinical (comorbidities, hospital length of stay and intubation), sociodemographic (sex, age, BMI, and socioeconomic position), and environmental (air pollution) factors. Preexisting comorbidities were associated with increased dyspnoea, fatigue and lower functional status at follow-up, in line with findings by a meta-analysis of long-term outcomes of COVID-19 [31], although available evidence still precludes us from determining the reasons for such association [31]. Severity of the acute disease has been implicated with persistent symptoms in other studies [7,8,35]. However, the underlying mechanism is not clear and likely to be multifactorial and include the direct effects of viral infection, immunological response, corticosteroid therapy and ICU stay [8]. Surprisingly, in our study we observed that intubation was associated with decreased anxiety and depression and decreased fatigue at follow up, which should be further explored in future studies.

Female sex was associated with all the selected outcomes examined. While mortality rates due to COVID-19 are higher in males [2-5], female sex has been reported as a risk factor for long-term symptoms in previous studies [7,8,36], and more specifically, associated with stress, depression, anxiety, fatigue, and dyspnoea [8]. The underlying mechanism for this finding is unknown, and likely multifactorial.

Although age was not associated with most outcomes examined in this study, younger patients were at higher risk of anxiety and depression at follow-up, in line with previous findings of a study focusing specifically on mental health in COVID-19 survivors [36]. Such a finding could be related to the impact of the pandemic on work availability, social isolation and other daily life impacts, as shown by a survey in China which found that younger people suffered a greater psychological impact during the pandemic [37]. While age is one of the most important predictors of the severity and lethality of COVID-19, there is no consensus about its role in the persisting symptoms [9,31,38].

We found a positive association between BMI and dyspnoea similar to a finding in a smaller German cohort [39]. BMI has been associated with worse outcomes in acute COVID-19, and possible mechanisms to explain our finding include more severe acute disease and worse overall health and exercise tolerance as BMI increases.

Race was not significantly associated with any outcome in our analysis. This contrasts with reports of disproportionate burden of infection and death from acute COVID-19 among African Americans and Hispanics in the US [40] and in Brazil [3].

Socioeconomic disparities have deeply affected the course of COVID-19 in Brazil with vulnerable regions suffering a disproportionate higher burden of morbidity and mortality [10,41]. We now provide novel information showing that socioeconomic conditions are also strong predictors of symptoms of Post-COVID-19 syndrome. The mechanisms behind greater susceptibility to infection and severe disease among deprived populations are likely to be the same involved in the persistence of symptoms. Chronic socioeconomic deprivation can impact overall health through different pathways including greater food insecurity, limited access to health care, and unhealthy lifestyles [42].

We have tested, by multilevel analysis, whether neighbourhood characteristics such as mean income, population density, percentage of green areas and air pollution levels could have an impact on different post COVID-19 symptoms. We observed that ambient levels of PM_2.5_ were associated with persistent dyspnoea, increased fatigue, and lower functional status at follow-up. To the best of our knowledge, this is the first study examining the role of air pollution in post-COVID-19 syndrome, although evidence is increasing on its role on the incidence and severity of this disease [14]. Air pollution is known to have acute and chronic systemic effects and particularly affects the lungs by mechanisms involving oxidative stress and inflammation, adversely affecting responses to viral infections [15,43]. A debilitated respiratory tract is prone to present more serious outcomes of COVID-19 [43] and thus, potentially predispose individuals to persisting symptoms. Although these potential mechanisms can explain the association between PM_2.5_ exposure and persistent dyspnoea, the association with fatigue and functional status is less clear. Thus, further studies may help us confirm this novel and important impact of air pollution on health.

The lack of effect of green areas on persistent symptoms could be related to the fact that we examined individual exposure based on the presence of green areas, but not the accessibility of individuals to these areas, an indicator probably more related to the expected beneficial effects of green areas on disease recovery. Similarly, population density might be important to enhance transmissibility and mortality [44] but not persistence of symptoms.

Our study has limitations. First, it was performed at a single academic hospital and, thus, results may not be generalizable. However, patients from this cohort were transferred from the entire Sao Paulo Metropolitan Region, which comprises 39 municipalities with 21 million inhabitants. Second, given that our hospital was the primary reference hospital for COVID-19 in the metropolitan area of Sao Paulo, the assessed patients possibly represented the most severe cases, hence, our results may not apply to less severe cases of COVID-19. In addition, subjects who agreed to participate in this study also had more severe acute disease and were more likely to have been admitted to the ICU and needed intubation than those who refused to participate. Therefore, patients included in our study probably represent a subset of more severe cases of COVID-19 patients who needed hospitalization and survived, and one should be cautious when extrapolating our findings to all COVID-19 survivors. Third, our findings are likely to be impacted by the higher burden of disease in LMICs and structural deficiencies of our health care system, and therefore may not be generalizable to other settings.

The study also has strengths, such as its large sample size and minimal missing data, which allows for more precise estimation of outcomes. In addition, we used standardized, validated instruments to detect symptoms, and objective tests whenever available. Patients were evaluated by a multidisciplinary team, allowing for more precise detection of symptoms. This cohort of patients will continue to be followed up and the multiple assessments already performed will be examined in greater depth, in order to confirm the present results, and thus, deepen the knowledge about Post-COVID-19 Syndrome. This will be important to better deal with the sequelae of this disease in the future.

## CONCLUSIONS

Through a multidisciplinary and integrated approach, we identified a high frequency of persistent symptoms 6 months after hospitalization in a large cohort of COVID-19 survivors. These persistent symptoms were associated with clinical, sociodemographic, and environmental variables.

With more than 200 million cases of the disease diagnosed worldwide, an increasing burden on health care systems, including mental health and rehabilitation services, is expected to occur, especially in megacities of LMICs, where structural problems in health care and high air pollution levels may pose additional challenges to the provision of health care for post-COVID syndrome.

## Additional material


Online Supplementary Document
