# Supplementary material for: Clinical, sociodemographic and environmental factors impact post-COVID-19 syndrome
Source: J Glob Health. 2022 Aug 9;12:05029. doi: 10.7189/jogh.12.05029 (PMC9359428; doi:10.7189/jogh.12.05029)
Supplement: Online Supplementary Document [file jogh-12-05029-s001.pdf]

Appendix 1

Clinical, sociodemographic and environmental factors impact post-COVID-19 syndrome

Table of contents

1. List of investigators -- 1
2. Supplementary Methods --- 3
3. Supplementary Tables -- 6
4. Supplementary Figures -- 15
5. Supplementary References -- 16

1. List of investigators

Juliana Carvalho Ferreira[1,2], MD; Tiana Carla Lopes Moreira[3,a], ScD; Adriana Ladeira de Araújo[4,a], PhD; Marta Imamura[5], MD; Rodolfo F. Damiano[6], MD; Michelle L. Garcia[1], PhD; Marcio V.Y. Sawamura[7], MD; Fabio R. Pinna[8], MD; Bruno F. Guedes[9], MD; Fabio Augusto Rodrigues Gonçalves[10], PharmD; Marcio Mancini[11], MD; Emmanuel Burdmann[12], MD; Demóstenes Ferreira da Silva Filho[13], PhD; Jefferson Lordello Polizel[14], PhD; Ricardo F. Bento[8,15], MD; Vanderson Rocha[16], MD; Ricardo Nitrini[9], MD; Heraldo Possolo de Souza[17], MD; Anna Sara Levin[18], MD; Esper Georges Kallas[18], MD; Orestes V. Forlenza[19], MD; Geraldo Busatto-Filho[4,6], MD; Linamara R. Batistella[5], MD; Carlos Roberto Ribeiro de Carvalho[1], MD; Thais Mauad[3,b], MD; Nelson Gouveia[20,b],PhD on behalf of the HCFMUSP Covid-19 Study Group*

[a] these authors contributed equally to this work

[b] these senior authors contributed equally to this work

HCFMUSP Covid-19 Study Group (corporate author)

* HCFMUSP COVID-19 Study Group: Tarcisio E.P. Barros-Filho, Edivaldo M. Utiyama, Aluisio C. Segurado, Beatriz Perondi, Anna Miethke-Morais, Amanda C. Montal, Leila Harima, Solange R.G. Fusco, Marjorie F. Silva, Marcelo C. Rocha, Izabel Marcilio, Izabel Cristina Rios, Fabiane Yumi Ogihara Kawano, Maria Amélia de Jesus, Juliana C. Ferreira,Carlos R R de Carvalho, Esper G Kallas, Anna Sara S. Levin, Heraldo Possolo de Souza, Maria Cristina Peres Braido Francisco, Carolina Mendes do Carmo, Clarice Tanaka, Maura Salaroli Oliveira, Thaís Guimarães, Carolina dos Santos Lázari, Marcello M.C. Magri, Julio F.M. Marchini, Alberto José da Silva Duarte, Ester Sabino, Silvia Figueiredo Costa

Affiliations (*HCFMUSP Covid-19 Study Group)*:

Hospital das Clinicas HCFMUSP, Faculdade de Medicina, Universidade de São Paulo, São Paulo, SP, Brasil.

Non-author members of the *HCFMUSP Long-COVID PASC Initiative:*

Members of this group contributed to outlining the components of the multidisciplinary assessment protocols, conducting assessments of patients and organizing the storage of demographic, clinical and biomarker data.

Claudia da Costa Leite, PhD; Cristiano Gomes, PhD; Euripedes C Miguel, PhD; Giovanni G. Cerri, PhD; Guilherme Fonseca; Jorge Hallak, PhD; José Eduardo Krieger, PhD; Luis Yu, PhD; Luiz Henrique Martins Castro, PhD; Maria Cassia J. Mendes Corrêa, PhD; Maria Elizabeth Rossi, PhD; Marilia Seelaender, PhD; Paulo A Lotufo, PhD; Roger Chammas, PhD; Rossana Pulcinelli Francisco, PhD; Thiago Avelino-Silva, PhD; Wilson Jacob Filho, PhD.

Affiliations (*HCFMUSP Long-COVID PASC Initiative)*:

Hospital das Clinicas HCFMUSP, Faculdade de Medicina, Universidade de São Paulo, São Paulo, SP, Brasil.

1. **Supplementary Methods**

Diagnosis of COVID-19

Hospital protocol for the diagnosis of COVID-19 was based on clinical history, tomographic finding of ground glass suggestive of COVID-19 plus confirmation with reverse-transcriptase polymerase chain reaction (RT-PCR) for SARS-CoV-2 on Abbott m200RT (Abbott Laboratories, Chicago, Illinois, USA) established at the Central Laboratory Division of HCFMUSP.

A confirmed case of COVID-19 was defined as a positive RT-PCR on swab, collected from nasopharyngeal and/or oropharyngeal samples, at admission with a minimum of 3 days of symptoms and, if negative, repeated after 48hours (1).

As of mid-April 2020, chemiluminescent immunoassays on Liaison XL analyzer (DiaSorin S.p.A., Saluggia, Italy) to detect serum antibodies became available in our Institution and were performed for highly suspect cases with at least 2 RT-PCR negative samples after seven days of the onset of symptoms. or in subjects with high clinical suspicion for whom a RT-PCR test was not available up to the 10th day of symptom onset (2).

If patients were admitted to the hospital as suspected COVID-19, but later had one or more negative RT-PCR and were diagnosed with other causes of respiratory failure, an infectious disease specialist reviewed the case to rule out COVID-19. When that happened, patients were transferred to other buildings in the hospital complex.

For this study, we included patients with confirmed COVID-19, defined as a positive RT-PCR or positive serologic test and clinical and tomographic findings compatible with COVID-19.

Patient care during hospital stay

Patient care was at the discretion of the ICU team, but the hospital developed institutional protocols specifically for COVID-19 patients, including the use of personal protective equipment, ventilatory management, thrombosis prophylaxis, oxygen support and use of antibiotics and corticosteroids. Specific drugs for treating COVID-19 were not recommended but could be used at the discretion of the attending physician. Dexamethasone was used for most patients after the publication of a clinical trial showing benefit in mid-June (3).

Assessments

Patients were invited to participate in the study by telephone by experienced research staff, followed by text messages when no answer was obtained after two telephone attempts. Those who accepted to participate were invited for in-person follow-up visits at six months when informed consent was obtained, and all study procedures were carried out. Invitations were made with the intent to evaluate patients within 3 weeks of the six-month after hospitalization mark. However, patients who agreed to participate but were unavailable at that window had their appointments rescheduled and were evaluated later.

In order to preserve the safety and social distancing of subjects and their relatives, all evaluations, except the radiological examinations, were conducted at one single sector at HCFMUSP, with each specialized team bringing a minimal number of researchers to work on site. Two separate facilities were used simultaneously for the multidisciplinary assessments of different subjects: one temporary outpatient center prepared to accommodate comfortably up to eight visits per day of subjects with a previous history of non-severe COVID-19, and the clinical research center of the Instituto do Coração (InCor-HCFMUSP), which accommodated up to ten subjects with a history of severe COVID-19 to be evaluated at each day.

During their visit, participants completed questionnaires, were submitted to physical examination and selected diagnostic tests, and provided blood samples. All study participants underwent four sets of interviews, with study coordinators, internal medicine specialists, psychiatrists, and specialists in rehabilitation.

Research coordinators registered selected items from the baseline interview of the Brazilian Longitudinal Study of Adult Health (ELSA-BRAZIL)(4) regarding socio-demographic characteristics, occupational history, as well as lifestyle habits.

Sociodemographic variables

Data collected included sociodemographic characteristics, occupational history, and lifestyle habits. Socioeconomic class was measured with a standardized questionnaire validated for the Brazilian population (5). This questionnaire assesses household assets, such as the number of bathrooms and automobiles in the household, access to public services, and educational level of the head of the family, to estimate the average family income per month. The classes are A (most affluent), B1, B2, C1, C2, D and E. For our analysis, we combined classes A, B1 and B2, which together comprise 36% of the population in the metropolitan area of Sao Paulo, and classes D and E, which together comprise 13% of the population. Class C corresponds to 52% of the population, according to data from 2019 (5). We refer to them as “high”, “medium” or “low” socioeconomic position.

Race was self-declared by participants, using the official Brazilian categories (white, mixed, black, Asian, indigenous).

We also collected information on population density and per capita income for each participant's neighborhood. These were obtained from a survey carried out by the metropolitan subway company (6) that is representative of the population of the metropolitan region of São Paulo. We divided the population of each neighborhood by its area to compute the population density and gathered data on average per capita income as an indicator of the socioeconomic conditions of the neighborhood.

Clinical assessments

We collected data about the hospitalization, including need for ICU admission, development of acute renal failure using the KDIGO classification (7) , intubation and duration of hospitalization Patients underwent physical examination and we registered comorbidities with the Charlson comorbidity index (8). Post COVID-19-symptoms were obtained with standardized scales, when available, or by direct question and a yes/no answer, were obtained. Table 1S lists all the symptoms evaluated. In order to detect COVID-19-related symptoms, we asked about the presence of the same symptoms before COVID-19, and report only symptoms that appeared or were significantly intensified after hospitalization for COVID-19.

Dyspnea was assessed using the Medical Research Council (MRC) dyspnea scale ranging from 0 to 5 (9). We considered the MRC to be abnormal when it was equal or greater than 2, (walks slower than contemporaries on level ground because of breathlessness or have to stop for breath when walking at own pace).

Functional status and fatigue were assessed with the Post-COVID-19 Functional Status (PCFS) Scale (10) and the Functional Assessment of Chronic Illness Therapy-Fatigue Scale (FACIT) (11), respectively. Values equal or above 2 in the PCFS and equal or below 39 in the FACIT scale were considered abnormal. Quality of life was measured with the EQ-5D scale (12) and we report the visual analog scale (VAS) component, ranging from 0 (worst possible health) to 100 (best possible health).

Psychiatrists interviewed participants using structured instruments for the detection of common mental disorders and assessments of severity of anxiety and depression using the hospital anxiety and depression scale (HADS)(13). Scores greater than 8 in either domain were considered as clinically relevant. Post-traumatic stress disorder (PTSD) was measured with the PTSD Checklist (14), and values above 30 were considered abnormal. Insomnia was measured with the insomnia severity index(15) and was considered abnormal for scores greater than 8. Memory impairment was measured with the memory complaint scale, (16). Body pain, loss of smell and loss of taste were measured with VAS (17,18). Methods for evaluating other symptoms are detailed in Table 1S.

A chest x-ray was obtained from all patients, and two thoracic radiologists independently classified it as normal or abnormal, with findings suggestive of COVID-19 related lesions, such as bilateral linear and reticular opacities (19). Patients completed a spirometry to assess lung function, performed according to Brazilian Thoracic Society standards (20). The forced vital capacity (FVC), as a percent of the predicted value, was the main variable of interest. Values below 80% of the predicted value were considered abnormal. Muscle strength was measured with the hand grip test (21) for both hands. Values below 25% percentile for age were considered abnormal. We measured oxygen saturation at rest by pulse oximetry and performed a 1-minute sit-to-stand test, while measuring pulse oximetry (22). Details of the instruments used are available in Table 1S.

Environmental variables

To estimate the exposure to greenspace and air pollution, each participant´s residential address was georeferenced and a 300m buffer area around each address was created. We used satellite images of the São Paulo metropolitan area from 2020, obtained from the U.S. Geological Survey – Earth Explorer (23) to classify and quantify the land covered by tree canopy. We used the 300m buffer for the tree canopy exposures according to the WHO recommendations, which corresponds to approximately 5 min walking distance along walkable roads or pathways (24).

A fusion of the multispectral bands of 8 meters and the panchromatic of 2 meters of the CBERS 4A satellite image resulted in a scene with 2 infrared and 2 meters resolution. The land cover classification was performed using the random forest algorithm (program QGIS2.18.11; Plugin Dtezaka). A detailed method of land cover classification is described elsewhere (25). Random forest (RF) is a robust learning classifier algorithm that is one of the most accurate methods of classifying land cover (adapted from(26). The following land cover classes were considered in this classification: tree canopy, grass, bare soil, cement floor, swimming pool, shade, roof (white, gray, dark, ceramic), asphalt, and river/lake (adapted from (26). For data analysis, the sum of tree canopies and grass was used as green space.

Air pollution data for 2018 (most recent year available) was obtained from the Atmospheric Composition Analysis Group of the Washington University in St Louis(27). Ground-level fine particulate matter ($PM_{2.5}$) was estimated using multiple satellite-based aerosol optical depth datasets combined with a chemical transport model, and subsequently calibrated to global ground-based observations using geographically weighted regression (28). Data were available as annual means ($\mu g/m^3$) in a gridded format with each grid cell representing 0.01 × 0.01 degrees, equivalent to 1.1 km × 1.1 km at the equator. We converted the value of each grid cell into points assigned to the geometric center of each cell (centroids). We calculated the annual mean $PM_{2.5}$ value in each 300m buffer area by averaging the values of each centroid contained within the buffer boundary. We used all composition $PM_{2.5}$ to better reflect the exposure of our study population (29).

Participants vs nonparticipants

Comparisons between patients who participated in this study (n=749) and those who did not participate because they refused to participate, could not be contacted or had any other exclusion criteria (n=930) (Figure 1) are shown in Table 2S. While the two subgroups were comparable regarding demographic characteristics, participants had higher BMI, more previous hypertension, longer duration of hospitalization, higher proportion of individuals who required ICU and intubation during hospitalization.

Supplementary Tables

Table 1S - Objective and subject symptoms and assessments performed during evaluation

Symptom	Instrument	Abnormal Cut off
**Standardized scales for symptoms**		
Fatigue	Functional Assessment of Chronic Illness Therapy-Fatigue (FACIT) Scale [11]	Score ≤ 39
Dyspnea	Medical Research Council (MRC) dyspnea scale [9]	Score ≥ 2
Memory impairment	Memory complaint scale (adapted for COVID-related complaint) [16]	Score ≥7
Depression	Hospital Anxiety and Depression Scale [13]	Score > 8 [9]
Anxiety	Hospital Anxiety and Depression Scale [13]	Score > 8 [9]
Post-traumatic stress	PTSD Checklist (enquiring about COVID-related symptoms) [14]	Score ≥ 30 [10]
Insomnia	Insomnia severity index [15]	Score ≥ 8[11]
Loss of smell	VAS [18]	≤ 80
Loss of taste	VAS [18]	≤ 80
Muscle / joint pain	VAS [17]	Score ≥ 65
**Subjective symptoms**		
Weakness	Direct "yes-no" questioning	Yes
Gait impairment	Direct "yes-no" questioning	Yes
Headache	Direct "yes-no" questioning	Yes
Paresthesia	Direct "yes-no" questioning	Yes
Dizziness	Direct "yes-no" questioning	Yes
Loss of consciousness	Direct "yes-no" questioning	Yes
Hearing loss	Direct "yes-no" questioning	Yes
Tinnitus	Direct "yes-no" questioning	Yes
Appetite loss	Direct "yes-no" questioning	Yes
Constipation /abdominal pain	Direct "yes-no" questioning	Yes
Diarrhea	Direct "yes-no" questioning	Yes
Nausea / vomiting	Direct "yes-no" questioning	Yes
Edema	Direct "yes-no" questioning	Yes
Nocturia	Direct "yes-no" questioning	Yes
Skin problems	Direct "yes-no" questioning	Yes
Cough	Direct "yes-no" questioning	Yes
Chest pain	Direct "yes-no" questioning	Yes
Nasal obstruction	Nasal obstruction symptom evaluation scale [29]	Yes to any question
Loss of concentration	Direct "yes-no" questioning	Yes
**General health status**		
Quality of life	VAS from the EQ-5D[12]	not applicable
Overall functional status	Post-COVID-19 Functional Status Scale [10]	Score ≥ 2

Footnote: (VAS) Visual analog scale. References for the scales used are shown in the supplementary reference list

Table 2S - Comparison between participants and survivors who did not participate

	Participants (n=749)	Non participants (n=930)	p-value
Age, mean ± SD, y	55 ± 14	56 ± 16	0·84
Male	397 (53%)	476 (51%)	0·46
Body Mass Index, median (IQR), kg/m^2	31 (27·5-36·6)	28 (23·7-33·5)	0·003
Hypertension	425 (57%)	466 (50%)	0·007
Cardiovascular disease	136 (18%)	137 (15%)	0·059
Diabetes	261 (35%)	301 (32%)	0·29
Duration of symptoms, median (IQR), d	8 (6 -11)	8 (5-11)	0·44
Duration of hospitalization, median (IQR), d	12 (7 - 23)	9 (6 - 16)	< 0·001
ICU stay	445 (59%)	394 (42%)	< 0·001
Need for intubation	305 (41%)	222 (24%)	< 0·001

Definition of abbreviations: SD: standard deviation; IQR: interquartile range; ICU: intensive care unit. Data are presented as counts (percentages), unless otherwise stated. P values were obtained with t-tests, Mann-Whitney test or chi square test, as applicable.

Table 3S - Prevalence of additional symptoms at follow up

Symptom	Prevalence
Dizziness	264 (36%)
Loss of concentration	208 (31%)
Nocturia	176 (24%)
Chest pain	143 (20%)
Cough	139 (19%)
Edema	129 (18%)
Nasal obstruction	118 (16%)
Paresthesia	116 (15%)
Skin problems	113 (15%)
Tinnitus	110 (15%)
Hearing loss	106 (14%)
Abdominal symptoms	101 (14%)
Weakness	96 (13%)
Appetite loss	91 (12%)
Gait problems	83 (11%)
Headache	80 (11%)
Diarrhea	44 (6%)
Loss of consciousness	27 (4%)
Nausea / vomiting	24 (3%)

Footnote. Data are presented as counts (percentages). Dizziness was missing for 11 participants; loss of concentration was missing for 79 participants; nocturia was missing for 19 participants; chest pain was missing for 23 participants; cough was missing for 14 participants; edema was missing for 18 participants; nasal obstruction was missing for 20 participants; skin problems was missing for 11 participants; tinnitus was missing for 12 participants; hearing loss was missing for 12 participants; abdominal symptoms was missing for 13 participants; appetite loss was missing for 10 participants; diarrhea was missing for 16 participants; loss of consciousness was missing for 18 participants; nausea / vomiting was missing for 13 participants.

Table 4S - Comparison of hospitalization characteristics of participants evaluated closer to the six-month mark and participants evaluated later

	Total (N=749)	≤ 200 days (N=384)	> 200 days (N=365)	p value
Age, years				0·76
-17-39	114 (16%)	57 (15%)	57 (17%)	
- 40 - 59	304 (42%)	155 (42%)	149 (43%)	
- > 60	301 (42%)	161 (43%)	140 (41%)	
Sex (male)	397 (53%)	202 (53%)	195 (54%)	0·83
Diabetes	261 (35%)	135 (35%)	126 (35%)	0·88
Hypertension	425 (57%)	233 (61%)	192 (53%)	0·03
Obesity	139 (19%)	67 (18%)	72 (20%)	0·45
Race*				0·95
- Black	102 (14%)	51 (13%)	51 (14%)	
- Mixed	273 (37%)	136 (37%)	137 (38%)	
- white	342 (47%)	176 (48%)	166 (46%)	
- Asian	10 (1%)	6 (2%)	4 (1%)	
- Indigenous	7 (1%)	4 (1%)	3 (1%)	
Education Level				0·32
- Education < 4 years	265 (36%)	140 (37%)	125 (35%)	
- Education 4-8 years	142 (19%)	75 (20%)	67 (19%)	
- Education 8-12 years	202 (27%)	102 (27%)	100 (28%)	
- Education > 12 years	134 (18%)	64 (17%)	70 (19%)	
Socioeconomic position				0·77
- A+B1+B2 (high)	196 (27%)	99 (26%)	97 (27%)	
- C1+ C2 (medium)	470 (64%)	244 (65%)	226 (63%)	
- D + E (low)	73 (10%)	34 (9%)	39 (11%)	
Charlson Score	3 (2, 4)	3 (2, 4)	3 (1, 4)	0·09
Intubation	305 (41%)	157 (41%)	148 (41%)	0·94
ICU admission	445 (60%)	224 (59%)	221 (61%)	0·55
Acute renal failure	315 (42%)	177 (47%)	138 (39%)	0·03
ICU LOS, median (IQR), d	10 (6, 18)	11 (6, 19)	9 (5, 16)	0·009
Hospital LOS, median (IQR), d	12 (7, 23)	14 (8, 25)	11 (6, 21)	0·002

Definition of abbreviations: ICU: intensive care unit; LOS: length of stay. Comparisons * The categories represent the Brazilian official race categories; Comparisons were made with Wilcoxon rank sum test, Fisher's Exact Test or trend test for ordinal variables, as appropriate.

Table 5S - Comparison between symptoms in participants who were evaluated closer to the six-month mark and participants evaluated later

	Total (N=749)	Return Within 200 days (N=384)	Return After 200 days (N=365)	p value
FVC, % predicted	84 (74, 94)	84 (72, 93)	86 (77, 94)	0·05
PCFS	1 (0, 2)	1 (0, 2)	1 (0, 2)	0·39
Handgrip, kgf	19 (10, 28)	19(10, 27)	19 (11, 28)	0·43
FACIT Score	42 (33, 47)	43 (34, 47)	41 (33, 47)	0·07
Anxiety	177 (26%)	85 (22%)	92 (32%)	0·01
Depression	146 (22%)	71 (19%)	75 (26%)	0·04
Dyspnea	219 (30%)	98 (26%)	121 (34%)	0·02
Fatigue	286 (38%)	135 (35%)	151 (42%)	0·10
Memory impairment	239 (35%)	118 (32%)	121 (38.2%)	0·08
Join/muscle Pain	299 (41%)	138 (37%)	161 (45%)	0·03
Insomnia	242 (32%)	112 (29%)	130 (36%)	0·07
PTSD	257 (35%)	141 (37%)	116 (33%)	0·28
Loss of taste	161 (23%)	75 (21%)	86 (25%)	0·21
Loss of smell	150 (21%)	60 (16%)	90 (25%)	0·002
Number of symptoms	2 (1, 5)	2 (1, 5)	2 (1, 5)	0·58

Definition of abbreviations: FVC: forced vital capacity; PCFS: post-COVID functional capacity; FACIT: Functional Assessment of Chronic Illness Therapy-Fatigue Scale; PTSD: Post-traumatic stress disorder. Comparisons were made with Wilcoxon rank sum test, Fisher's Exact Test or trend test for ordinal variables, as appropriate.

Table 6S – Univariate regression estimates for sociodemographic, clinical and environmental factors associated with selected symptoms and scales in patients with Covid-19 at follow-up

		Dyspnea		Fatigue		Functional Status		Anxiety/Depression	
		**Estimate (95%CI)**	**p-value**	**Estimate (95%CI)**	**p-value**	**Estimate (95%CI)**	**p-value**	**Estimate (95%CI)**	**p-value**
Sex	female	-	-	-		-		-	-
	male	-0·47(-0·62 – -0·32)	**<0·001**	4·88(3·53 – 6·23)	**<0·001**	-0·40(-0·56 – -0·24)	**<0·001**	-5·10 (-6·33 – -3·87)	**<0·001**
Age		0·00(-0·00 – 0·00)	0·18	0·00(-0·04 – 0·04	0·99	0·00(0·00 – 0·01)	**0·002**	-0·06(-0·10 – -0·01)	**0·008**
Race	White	-	-	-	-	-	-	-	-
	Black	0·06(-0·17 – 0·30)	0·59	-0·78(-2·93 – 1·36)	0·47	0·08(-0·16 – 0·33)	0·49	0·40(-1·59 – 2·39)	0·69
	Mixed	0·11(-0·06 – 0·28)	0·2	0·05(-1·49 – 1·60)	0·95	0·08(-0·09 – 0·26)	0·37	-0·09(-1·52 – 1·34)	0·90
	Asian	0·22(-0·45 – 0·91)	0·52	2·49(-3·62 – 8·60)	0·42	0·23(-0·47 – 0·93)	0·52	-1·85(-7·52 – 3·81)	0·52
	Indigenous	-0·07(-0·88 – 0·74)	0·86	-0·49(-7·77 – 6·78)	0·89	-0·31(-1·15 – 0·52)	0·47	1·24(-5·50 – 7·99)	0·72
Social Class	A+B	-	-	-	-	-	-	-	-
	C	0·37(0·19 – 0·55)	**<0·001**	-0·92(-2·53 – 0·69)	0·26	0·20(-0·37 – 2·60)	**0·03**	1.11(-0.37 – 2.60)	0·14
	D-E	0·71(0·42 – 1·00)	**<0·001**	-3·75(-6·35 – -1·14)	**0·005**	0·55(0·69 – 5·48)	**<0·001**	3.08(0.69 – 5.48)	**0·01**
Education	< 4 years	-	-	-	-	-	-	-	-
	4 -8 years	-0·07(-0·29 – 0·14)	0·52	-0·72(-2·70 – 1·25)	0·47	-0·09(-0·32 – 0·13)	0·41	0·48(-1·34 – 2·31)	0·60
	8-12 years	-0·17(-0·37 – 0·02)	**0·09**	-0·27(-2·05 – 1·50)	0·76	-0·29(-0·50 – -0·09)	**0·004**	0·49(-1·15 – 2·13)	0·56
	>12years	-0·23(-0·46 – -0·01)	**0·04**	0·89(-1·11 – 2·91)	0·38	-0·28(-0·51 – -0·05)	**0·016**	0·73(-1·13 – 2·60)	0·44
Charlson score		0·06 (0·02 – 0·11)	**0·002**	-0·57(-0·96 – -0·17)	**0·004**	0·10 (0·05 – 0·14)	**<0·001**	-0·20(-0·57 – 0·15)	0·26
Body mass Index		0·01(0·00 – 0·02)	**0·003**	-0·10(-0·20 – -0·01)	**0·02**	0·00 (-0·00 – 0·01)	0·17	0·09(0·00 – 0·17)	**0·03**
Smoking		0·11(-0·04 – 0·27)	0·16	-0·40(-1·84 – 1·03)	0·58	0·13 (-0·03 – 0·29)	0·11	0·16(-1·16 – 1·48)	0·81
Intubation	No	-	-	-	-	-	-	-	-
	Yes	-0·06(-0·22 – 0·09)	0·41	1·20(-0·21 – 2·61)	0·09	0·11(-0·05 – 0·27)	0·18	-1·58(-2·88 – -0·27)	**0·017**
Acute renal failure		0·07 (-0·24 – 0·40)	0·64	-1·19 (-4·08 – 1·69)	0·42	0·01 (-0·32 – 0·34)	0·94	-0·24 (-2·87 – 2·37)	0·85
Duration of hospitalization		0·00 (-0·00 – 0·00)	0·69	-0·01(-0·05 – 0·01)	0·29	0·00 (0·00 – 0·01)	**<0·001**	-0·02 (-0·06 – 0·00)	0·12
Air Pollution		0·13(0·00 – 0·25)	**0·036**	-0·94(-2·05 – 0·16)	0·09	0·104 (-0·02 – 0·23)	0·11	0·09 (-0·93 – 1·11)	0·86
Greenspace		-0·00(-0·00 – 0·00)	0·82	-0·00 (-0·04 – 0·04)	0·94	-0·00(-0·00 – 0·00)	0·53	-0·00(-0·04 – 0·04)	0·97
Per capita income		-0·00 (-0·00 – 0·00)	**0·02**	-0·00 (-0·00 – 0·00)	0·10	-0·00 (-0·00 – 0·00)	0·13	-0·00 (-0·00 – 0·00)	0·33
Population density		-0·00 (-0·00 – 0·00)	0·85	0·00 (-0·00 – 0·01)	0·24	0·00 (-0·00 – 0·00)	0·46	0·00 (-0·00 – 0·00)	0·84

Footnote: 95% IC: 95% confidence interval. We collapsed socioeconomic position categories A+B1+B2 (high), C1+C2 (medium) and D+E (low) for the present analysis. Estimates are coefficients from the multilevel regression. P values obtained with a multilevel model.

Supplementary Figures

Figure 1S –number of symptoms for each participant at follow up

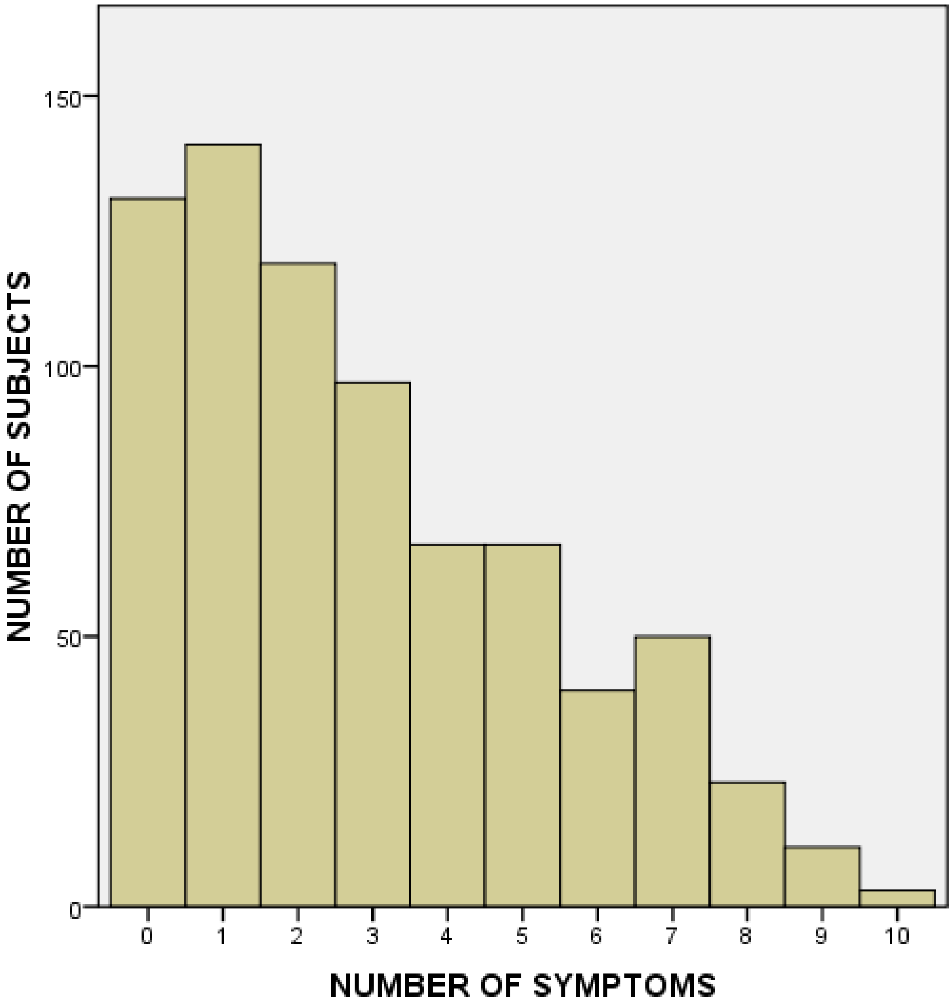

Footnote: histogram of the number of symptoms, out of the ten symptoms measures with standardized scales

References

References

1. Corman VM, Landt O, Kaiser M, Molenkamp R, Meijer A, Chu DK, et al. Detection of 2019 novel coronavirus (2019-nCoV) by real-time RT-PCR. Eurosurveillance. 2020;25:2000045.

2. Lisboa Bastos M, Tavaziva G, Abidi SK, Campbell JR, Haraoui L-P, Johnston JC, et al. Diagnostic accuracy of serological tests for covid-19: systematic review and meta-analysis. BMJ. 2020;370:m2516.

3. RECOVERY Collaborative Group, Horby P, Lim WS, Emberson JR, Mafham M, Bell JL, et al. Dexamethasone in Hospitalized Patients with Covid-19. N Engl J Med. 2021;384:693-704.

4. Aquino EML, Barreto SM, Bensenor IM, Carvalho MS, Chor D, Duncan BB, et al. Brazilian Longitudinal Study of Adult Health (ELSA-Brasil): Objectives and Design. Am J Epidemiol. 2012;175:315–24.

5. Critério Brasil - ABEP [Internet]. Socioeconomic class in Brazil. ABEP Brasil. Available: CCEB 2020 - PNADC 2019 https://www.abep.org/criterio-brasil. 2021. Accessed 4 October 2021.

6. COMPANHIA DO METROPOLITANO DE SÃO PAULO - METRÔ. Resultados da Pesquisa Origem e Destino 2017 [Internet]. São Paulo; 2019. Available: http://www.metro.sp.gov.br/pesquisa-od/arquivos/Ebook%20Pesquisa%20OD%202017_final_240719_versao_4.pdf. Accessed 4 October 2021.

7. Kellum JA, Lameire N, Aspelin P, Barsoum RS, Burdmann EA, Goldstein SL, et al. Kidney disease: Improving global outcomes (KDIGO) acute kidney injury work group. KDIGO clinical practice guideline for acute kidney injury. Kidney Int Suppl. 2012;2:1–138.

8. Charlson ME, Pompei P, Ales KL, MacKenzie CR. A new method of classifying prognostic comorbidity in longitudinal studies: Development and validation. J Chronic Dis. 1987;40:373–83.

9. Bestall JC, Paul EA, Garrod R, Garnham R, Jones PW, Wedzicha JA. Usefulness of the Medical Research Council (MRC) dyspnoea scale as a measure of disability in patients with chronic obstructive pulmonary disease. Thorax. 1999;54:581–6.

10. Klok FA, Boon GJAM, Barco S, Endres M, Geelhoed JJM, Knauss S, et al. The Post-COVID-19 Functional Status scale: a tool to measure functional status over time after COVID-19. Eur Respir J. 2020;56:2001494.

11. Webster K, Cella D, Yost K. The Functional Assessment of Chronic Illness Therapy (FACIT) Measurement System: properties, applications, and interpretation. Health Qual Life Outcomes. 2003;1:79.

12. Rabin R, de Charro F. EQ-5D: a measure of health status from the EuroQol Group. Ann Med. 2001;33:337–43.

13. Zigmond AS, Snaith RP. The Hospital Anxiety and Depression Scale. Acta Psychiatr Scand. 1983;67:361–70.

14. Weathers FW, Litz BT, Herman DS, Huska JA, Keane TM. The PTSD Checklist (PCL): Reliability, validity, and diagnostic utility. In: annual convention of the international society for traumatic stress studies, San Antonio, TX. San Antonio, TX; 1993. Available: www.ptsd.va.gov. Accessed 4 October 2021.

15. Bastien C. Validation of the Insomnia Severity Index as an outcome measure for insomnia research. Sleep Med. 2001;2:297–307.

16. Vale FAC, Balieiro-Jr AP, Silva-Filho JH. Memory complaint scale (MCS): Proposed tool for active systematic search. Dement Neuropsychol. 2012;6:212–8.

17. McCormack HM, Horne DJ, Sheather S. Clinical applications of visual analogue scales: a critical review. Psychol Med. 1988;18:1007–19.

18. Brandão Neto D, Fornazieri MA, Dib C, Di Francesco RC, Doty RL, Voegels RL, et al. Chemosensory Dysfunction in COVID-19: Prevalences, Recovery Rates, and Clinical Associations on a Large Brazilian Sample. Otolaryngol Neck Surg. 2021;164:512–8.

19. Litmanovich DE, Chung M, Kirkbride RR, Kicska G, Kanne JP. Review of Chest Radiograph Findings of COVID-19 Pneumonia and Suggested Reporting Language. J Thorac Imaging. 2020;35:354–60.

20. Pereira CA de C, Sato T, Rodrigues SC. New reference values for forced spirometry in white adults in Brazil. J Bras Pneumol. 2007;33:397–406.

21. Bohannon RW. Muscle strength: clinical and prognostic value of hand-grip dynamometry. Curr Opin Clin Nutr Metab Care. 2015;18:465–70.

22. Bohannon RW, Crouch R. 1-Minute Sit-to-Stand Test: systematic review of procedures, performance, and clinimetric properties. J Cardiopulm Rehabil Prev. 2019;39:2–8.

23. The U.S. Geological Survey – Earth Explorer. EarthExplorer [Internet]. Earth Explorer. Available: https://earthexplorer.usgs.gov/. Accessed 4 October 2021.

24. World Health Organization. Urban green spaces and health. World Health Organization. Regional Office for Europe; 2016. [cited 2020 Jan 20]. Available: http://www.euro.who.int/__data/assets/pdf_file/0005/321971/Urban-green-spaces-and-health-review-evidence.pdf?ua=1. Accessed 4 October 2021.

25. Moreira, Polizel, Santos, Filho, Bensenor, Lotufo, et al. Green Spaces, Land Cover, Street Trees and Hypertension in the Megacity of São Paulo. Int J Environ Res Public Health. 2020;17:725.

26. Myeong S, Nowak DJ, Hopkins PF, Brock RH. Urban cover mapping using digital, high-spatial resolution aerial imagery. Urban Ecosyst. 2001;5:243–56.

27. Atmospheric Composition Analysis Group | Washington University in St. Louis [Internet]. [cited 2021 Oct 25]. Available: https://sites.wustl.edu/acag/. Accessed 4 October 2021.

28. Hammer MS, van Donkelaar A, Li C, Lyapustin A, Sayer AM, Hsu NC, et al. Global Estimates and Long-Term Trends of Fine Particulate Matter Concentrations (1998–2018). Environ Sci Technol. 2020;54:7879–90.

29. van Donkelaar A, Martin RV, Brauer M, Hsu NC, Kahn RA, Levy RC, et al. Global Estimates of Fine Particulate Matter using a Combined Geophysical-Statistical Method with Information from Satellites, Models, and Monitors. Environ Sci Technol. 2016;50:3762–72.
